# Development and evaluation of the Acromegaly Symptom Diary

**DOI:** 10.1186/s41687-023-00541-7

**Published:** 2023-02-15

**Authors:** Susan Martin, Randall H. Bender, Alan Krasner, Tonya Marmon, Michael Monahan, Lauren Nelson

**Affiliations:** 1 Patient-Centered Outcomes Assessment, RTI Health Solutions, Ann Arbor, MI USA; 2 Patient-Centered Outcomes Assessment, RTI Health Solutions, 200 Park Offices Drive, Research Triangle Park, NC 27709 USA; 3 Crinetics Pharmaceuticals, Inc., San Diego, CA USA; 4 Marmon Biostatistics, Fair Oaks, CA USA

**Keywords:** Acromegaly Symptom Diary (ASD), Qualitative interviews, Psychometric evaluation, Quality of life, Patient-reported outcomes, Growth hormone excess, IGF-I, ACROBAT

## Abstract

**Background:**

Patient-reported outcome (PRO) measures are important to consider when evaluating treatments, yet there are no PRO measures for patients with acromegaly that have been developed in accordance with US Food and Drug Administration guidance. Acromegaly is a rare, chronic condition caused by hypersecretion of growth hormone. Disease activity is monitored by measurement in serum of growth hormone and insulin-like growth factor-I. The objectives of this research were to develop the Acromegaly Symptom Diary (ASD), establish a scoring algorithm, and evaluate the psychometric measurement properties of the ASD.

**Methods:**

Semistructured interviews consisting of concept elicitation and cognitive debriefing components were conducted with 16 adult participants with acromegaly. The concept elicitation component identified symptoms important to individuals with acromegaly. The cognitive debriefing component gathered information about the participants’ experience with each proposed item of the ASD, their thought process for answering each question, and their interpretation of the items. The psychometric properties of the draft ASD were then evaluated using data from the ACROBAT Evolve (NCT03792555; n = 13) and ACROBAT Edge (NCT03789656; n = 47) clinical trials.

**Results:**

The 16 participants from the interviews described ongoing symptoms, with the most frequently reported being joint pain (n = 13) and fatigue (n = 12), followed by swelling (n = 8), headache (n = 7), and mood swings (n = 6), and were able to interpret and understand the ASD items and had no issues with the 24-hour recall period. From data collected in the clinical studies, the psychometric properties of internal consistency (0.91 − 0.80), test-retest reliability with item-level and total ASD scores (> 0.70), baseline construct validity (r ≥ |0.38|) across scales, and responsiveness to change (r = 0.52–0.56) were supported for the ASD. The proposed preliminary threshold range to characterize a meaningful change from the patients’ perspective for the ASD total is a 4- to 6-point change for improvement or worsening out of a total score of 70.

**Conclusion:**

These findings provide qualitative and quantitative evidence to support the ASD as fit for the purpose of evaluating the symptom experience of patients with acromegaly in clinical trials.

**Supplementary Information:**

The online version contains supplementary material available at 10.1186/s41687-023-00541-7.

## Background

Acromegaly is a rare, chronic condition caused by hypersecretion of growth hormone (GH), usually due to a pituitary adenoma, which in turn causes elevated circulating levels of insulin-like growth factor I (IGF-I) [1]. Additional complications of uncontrolled acromegaly include hypertension, diabetes mellitus, sleep apnea, carpal tunnel syndrome, and arthritis, as well as early mortality related to cardiovascular disease from long-term elevation of circulating GH and IGF-I levels [1–4]. Soft-tissue overgrowth results in a distinct phenotype characterized by changes in appearance, including coarsening and thickening of facial features [1]. Gigantism is the consequence of excess GH prior to fusion of the epiphyseal plates, resulting in tall stature and features of acromegaly [5]. In adults, the presenting symptoms of acromegaly are not specific and can include lethargy, headache, increased sweating, and acral/soft-tissue changes [6]. The typical first-line treatment is surgical removal of the tumor, but if hypersecretion of GH and IGF-I continues, then symptoms are commonly managed with pharmacological treatments, such as somatostatin receptor ligands (SRL) (e.g., octreotide, lanreotide) or other medications that either reduce GH hormone secretion or antagonize the GH receptor [1, 7].

Patient-reported outcome (PRO) measures that assess individuals’ perspective of symptom burden, treatment satisfaction, and quality of life (QOL) are important to consider when evaluating treatments [7, 8]. The United States (US) Food and Drug Administration (FDA) has specific guidance for the development of PRO measures, which includes defining a conceptual framework for a measure, identifying an endpoint model, and establishing content validity [9, 10]. Currently available acromegaly PRO measures include the Acromegaly Quality of Life Questionnaire (AcroQoL) [11], the Acromegaly Treatment Satisfaction Questionnaire (Acro-TSQ) [8], and the Patient-assessed Acromegaly Symptom Questionnaire (PASQ) [12]. A recent systematic literature review on PRO measures for acromegaly [13] reported that the PASQ, as well as most other PRO measures used for acromegaly, have not been validated. While the AcroQoL and the Acro-TSQ are supported by psychometric evaluation research [8, 11, 14, 15], the unspecified recall period of the AcroQoL and the variable recall period of the Acro-TSQ are not aligned with FDA guidance [9, 10] that PRO measures have a specified recall period that occurs over a short time period when responses rely on a participant’s memory. To our knowledge, there are currently no PRO measures specific to acromegaly symptoms that were developed based on this FDA guidance.

The primary objective of this research was to develop the Acromegaly Symptom Diary (ASD). Secondary objectives included establishing a scoring algorithm and evaluating the psychometric measurement properties of the ASD. The anticipated context of use for the ASD is in future acromegaly clinical trials that evaluate new treatments.

## Methods

### Development and evaluation of the ASD

A targeted literature review was conducted to identify existing acromegaly assessments. Semistructured interviews of 60 min each were then scheduled with eligible individuals who were recruited through the Acromegaly Community patient advocacy group using a purposive sampling approach. To be included in the study, participants needed to be aged 18–75 years; able to speak English; diagnosed with acromegaly; on a stable dose for at least 3 months of either an SRL, dopamine agonist, or pegvisomant as monotherapy or in combination; and still experiencing symptoms of acromegaly. This study was approved by the RTI Institutional Review Board, and all participants provided verbal informed consent that was audio recorded. Each interview was conducted by two experienced researchers, lasted approximately 60 min, and was recorded and transcribed. Transcripts were verified through an iterative process of technical and editorial review.

The interviews consisted of two parts: concept elicitation and cognitive debriefing. The purpose of these interviews was to understand and document the key symptoms of acromegaly and those symptoms likely to improve with treatment from a patient perspective. Additionally, we evaluated the extent to which the existing items included in the ASD comprehensively capture the most important symptoms to patients. The findings can be used to indicate if the ASD is relevant to the experience of patients with acromegaly and, in doing so, provide evidence in support of the content validity of the ASD. The concept elicitation portion was conducted to identify symptoms (both past and present) important to individuals with acromegaly. Questions were open-ended to allow participants to describe their experiences with acromegaly freely (e.g., date of diagnosis, symptoms, diagnosis methods). The cognitive debriefing portion of the interview was designed to gather information about the participants’ experience with each proposed item of the ASD, their thought process for answering each question, and their interpretation of the items.

#### Data collection and analyses

During each interview, one researcher served as the primary interviewer, while the other researcher took notes and monitored the need for additional questions or probes. All interviews began with open-ended questions to encourage participants to discuss their experiences with acromegaly (e.g., date of diagnosis, symptoms, diagnosis methods). Participants were also asked to report on the acromegaly symptoms (both current and previous) that they experienced, as well as to identify the symptoms that were most bothersome to them. After the initial portion of the interview, participants were asked to engage in cognitive debriefing of the draft ASD. A “think aloud” format was used to gather information about participants’ interpretations of each item and about the process they used to develop each response.

Thematic analysis was used to identify and document the spontaneous and probed concepts described during the concept elicitation interviews and to provide evidence of concept saturation. Quotes representative of participant feedback are presented in this paper to illustrate the key symptoms. For the cognitive debriefing results, we conducted an analysis of the field notes to identify any potential problems within the questionnaire based on participant feedback. Specifically, we reviewed the results of interviews to identify and summarize patterns in the way participants interpreted and responded to each item and to determine how well the items captured concepts relevant to the participants. All analyses were conducted using Microsoft Excel and Word.

### Psychometric evaluation

The psychometric properties of the ASD were evaluated using pooled data from two clinical trials, ACROBAT Evolve [16] (NCT03792555; n = 13) and ACROBAT Edge [17, 18] (NCT03789656; n = 47). ACROBAT Evolve was a phase 2, double-blind, placebo-controlled, multicenter, randomized withdrawal study to evaluate the safety, pharmacokinetics, and efficacy of paltusotine in patients with an IGF-I within the age-related reference range while on SRL therapy in the form of octreotide long-acting release (LAR) or lanreotide depot. ACROBAT Edge was a phase 2, single-arm study that enrolled patients with acromegaly who switched to paltusotine from SRL-based therapy. The primary endpoint was change from Baseline to Week 13 in IGF-I for patients with elevated IGF-I levels (between 1 and 2.5 times the upper limit of normal for the age-related reference range) while on octreotide or lanreotide monotherapy (n = 25). ACROBAT Evolve included more than 45 centers in the US, Europe, South America, and Oceania.

In both trials, patients underwent a screening period of 4–6 weeks (i.e., Screening Visit of up to three visits) and then received 13 weeks of treatment (i.e., Week 1-Week 13) with once-daily oral paltusotine (10 mg/day, titrated as necessary to a maximum 40 mg/day). The end of treatment (EOT) at the end of Week 13 was followed by a 4-week wash-out period (i.e., Weeks 14–17). Details about key timepoints for each measure are presented in Table S-1 (Additional file 1).

### Measures

The measures used in this psychometric evaluation included the ASD, the AcroQoL [11], Patient Global Impression of Severity (PGI-S) [19], Patient Global Impression of Improvement (PGI-I) [19], and the EQ visual analogue scale (EQ-VAS) [20]. The draft ASD was developed following the completion of the concept elicitation and cognitive debriefing interviews. This 9-item PRO measure assessed symptoms associated with acromegaly, including headache, joint pain, sweating, fatigue, leg weakness, swelling, numbness/tingling, sleep difficulties, and short-term memory difficulties. The symptoms experienced in the last 24 h were rated on an 11-point numeric scale ranging from 0 (no symptom) to 10 (worst symptom). The AcroQoL is a disease-specific questionnaire consisting of 22 items measured on a 5-point Likert scale assessing frequency of occurrence (always [1] to never [5]) or degree of agreement (completely agree [1] to completely disagree [5]) with the statements [11]. A total score was calculated as the sum of the 22 items, ranging from 22 (worst QOL) to 110 (best QOL). The PGI-S is a single question asking for an overall rating of current acromegaly symptom severity, scored as 0 (none) to 3 (severe) [19]. The PGI-I is a single question asking for overall acromegaly symptom change during the study compared with Baseline, scored as − 3 (very much improved) to 3 (very much worse) [19]. The EQ-VAS asks participants to rate their global health state by drawing a line on the scale between the labels “best imaginable health state” (100) and “worst imaginable health state” (0) [20]. Additionally, GH and IGF-I were measured as per the ACROBAT study protocols.

### Analyses

All psychometric analyses were planned in accordance with the recommendations outlined in the FDA PRO guidance [9, 10, 21]. Item-level response frequencies were generated to show how many participants completed the ASD daily for the 1- to 7-day Baseline and wash-out periods. Sleep Difficulties (Item 8) and Short-Term Memory (Item 9) items were not included in the calculation of the ASD item total score per a recommendation from the FDA due to these two items being considered “impact” items rather than key symptoms of acromegaly. Accordingly, the analyses were based on ASD total scores ranging from 0 to 70 (Items 1–7) rather than 0 to 90, with a higher score indicating a worse state. The total ASD scores were derived from daily ASD scores where a weekly average was defined as the sum of a scored item over the course of a week divided by the number of days on which the item was completed. While ASD Items 8 and 9 are not included in the total score, we have presented individual analyses for these two items. For each ASD item, at least four completed scores were needed to generate the ASD total score (consecutive and nonconsecutive scores, as well as 1–3 missed days allowed). Inter-item correlations (IICs) and internal consistency analyses were conducted to evaluate the scoring of the ASD, where a Cronbach’s coefficient alpha ˃0.70 was used as the cut-off value for evaluation [22, 23].

Test-retest reliability of the ASD total scores was assessed with intraclass correlation coefficients (ICCs) based on two-way mixed-effects analysis of variance (ANOVA) models for absolute agreement [24], where evaluation of the ICCs followed the recommended threshold of 0.70 to be considered stable across time [25]. The assessments were conducted from the first two consecutive pairs of timepoints when a patient had the same PGI-S rating and non-missing scores on the corresponding ASD scores within the screening period (i.e., Visits 1b and 2, 1b to 3, and 2 and 3). Correlations between the ASD total scores and supporting measures PGI-S, AcroQoL, and EQ-VAS were conducted to examine convergent and discriminant ability using Cohen’s guidelines [26]. *A priori* hypotheses for construct validity are presented in Table S-2 (Additional file 1).

The values from the PGI-S and IGF-I measures were used as an indication of a patient’s status on supporting measures to form a set of known groups. The ASD item and total scores were then compared between groups derived from the PGI-S responses (none to severe) and groups based on serum IGF-I levels (normal range, above normal range). The ANOVA models were used to examine differences in the mean ASD total score by patients classified on the basis of these supporting measures at Baseline and Follow-up Week 17. The ability to detect change, or responsiveness, refers to the extent to which an instrument can detect changes in patients’ clinical status. The potential for the ASD total score to detect change in patient-reported acromegaly symptoms was examined via ANOVA models by the change in PGI-S responses status and correlations between ASD total change scores and the supporting measures. ANOVA models were used to examine the mean ASD total change across levels of change in the supporting measures. Change from Screening Visit to Baseline was selected on the basis of results from a study of 195 patients with acromegaly [7] in which 52% of the patients reported worsening of symptoms in the days before receiving their next treatment. Change from EOT to Follow-up Week 17 was selected because patients were off treatment during the Follow-up period, and therefore, change was anticipated.

Anchor and distribution-based methods were used to explore meaningful within-person change thresholds (improvement or worsening) for the ASD total score. Given the small study sample size, the initial threshold for the ASD weekly total score was estimated on the basis of change scores from EOT (Week 13) to the end of the wash-out period (Week 17) in the data from the two clinical trials. The PGI-S was selected as the primary anchor by which the pattern of ASD total change scores from EOT to Follow-up Week 17 across different change levels of PGI-S was assessed using the minimum responsiveness correlation of 0.371 recommended for anchor measures [27–29]. The distribution-based methods described in the FDA PRO guidance [9] were applied to examine ASD responder definitions from a measurement effect size and precision perspective. We computed the value for a half standard deviation (SD) of Baseline ASD scores and the standard error of measurement (SEM) using the SD of the ASD scores at Baseline and the test-retest reliability estimate of the ASD scores. The half SD is a commonly used, distribution-based method to define minimally important differences in clinical outcome assessment research [30–32]. Because the SEM includes reliability, it explicitly considers measurement precision and has been shown to be relatively stable across populations [33].

## Results

### Development and evaluation of the ASD

A total of 16 individuals with acromegaly participated in the development and evaluation of the ASD. The average participant was aged 47 years (range, 18–75 years), White (n = 15), and female (n = 11). Participants had acromegaly, on average, for 9 years. Approximately 56% of participants resided in the Southeast region of the US. No participants were naive to treatment, as all were taking stable doses of either octreotide, lanreotide, pegvisomant, or cabergoline for at least 3 months for treatment of acromegaly symptoms (Table 1).

**Table 1:** Participant characteristics for the concept elicitation and cognitive debriefing interviews

Characteristic	Total (N = 16)
Age, mean (range), years	47.3 (24–63)
Sex, n (%)	
Male	5 (31.3)
Female	11 (68.8)
Race/ethnicity, n (%)	
White, not Hispanic	15 (93.8)
Hispanic	1 (6.3)
Highest level of education, n (%)	
High school/Secondary school diploma/GED	4 (25)
Partial college	4 (25)
College/Baccalaureate degree	7 (43.8)
Graduate degree	1 (6.3)
Employment, n (%)	
Full-time	3 (18.8)
Part-time	1 (6.3)
Unemployed/disabled	12 (75)
Years since diagnosis, mean (range)	9.1 (2–22)
Medication, n (%)	
Octreotide	8 (50.0)
Lanreotide	7 (43.8)
Pegvisomant	5 (31.3)
Cabergoline	2 (13.0)

GED = General educational development; US = United States

For the concept elicitation portion, most of the 16 participants described initially noticing an increase in body weight and specific body parts changing in size before receiving their official diagnosis. The most frequently reported spontaneous symptoms included joint pain (n = 13), fatigue (n = 12), sign of swelling (n = 8), symptoms of headaches (n = 7), and mood swings (n = 6) (Table 2). Upon participants being asked which of these symptoms and signs improved with treatment, a similar pattern emerged, with joint pain again being the most often reported (n = 5), followed by fatigue (n = 4), headaches (n = 4), swelling (n = 3), and sweating (n = 3). Notably, only 1 of the 6 participants that reported mood swings indicated this symptom was helped by treatment. Several participants described the effect of treatment on their symptoms:

**Table 2:** Symptoms and signs of acromegaly spontaneously reported

Signs/symptoms	Participant quotes
Joint pain	*I’ve put on X amount of weight and have grown excessively and quickly, how that strain would…it’d be like stretching something and it doesn’t really go back to the way it was, it just kind of is…disfigured. And I know, obviously, I’m not that disfigured, but, you know, that pain is there from the act of being metaphorically stretched. (ID 14)*
	*For my hips and my joints, I’m attributing it to the acromegaly. The thinning of the cartilage in between my joints because of the thickening on the ends of my joints of my bones, the bones are spurs wearing down the cartilage in between. The joint pain is severe. (ID 1)*
	*The things I noticed is definitely joint pain. Like usually it gets worse. Like my lower back, my knees will ache. I’m actually, I’m starting to have problems with my one hip…is starting to, when I stand up and I move, it’s starting to get sore. So I don’t know what that’s all about, but it all kind of comes and goes. (ID 2)*
Fatigue	*The fatigue thing is definitely something that bothers me, and it’s some days, it’s worse than the others. I tell my wife sometimes it’s, it’s like I get up, I help my wife get my kids off to school, and there are some days it’s very real. Like it’s not a, ‘Oh, I had, I didn’t sleep good last night.‘ It’s a total lack of energy. (ID 2)*
	*It’s [fatigue] just so chronic, it’s every day. It’s just a question of degrees, really. (ID 3)*
	*…you get real tired. The fatigue is there. You don’t have hardly any…and if it’s warm outside, it seems like in an hour you’re just drained because I try to walk to my mailbox up there for a form of exercise, which it is quite a ways. You know, it’s probably quarter mile up the hill. But by the time I get back, I pretty much need a nap. [laughter] I don’t have any energy anymore. (ID 4)*
Sign of swelling	*…when they [hands] swell really bad, my hand hurts, my knuckles hurt. And then, not being able to put on my wedding band, things like that, it’s frustrating. (ID 11)*
	*It [swelling] hurts. I can’t even walk like around in the store. I have to sit, you know, in the electric cart. (ID 8)*
	*My hands and feet start to swell to the point that, my worse ones, that I have to wear flip-flops because my tennis shoes don’t fit. My rings have to come off. Uh, it’s hard to close my hands or grip something tightly because of the pain the swelling has caused in my fingers. (ID 14)*
Symptoms of headaches	*The pain, you learn, you know, you learn to deal with it. It’s the new normal and most, most days I can deal with it without medication. I think the hardest part is the mental part. (ID 5)*
	*I was having severe headaches, and the headaches had gotten worse and worse over a few months, and it started affecting my vision. Like what…so it ended up getting to a point where I think I was in so much pain, I was in agony, and I couldn’t take it anymore. (ID 2)*
	*We get these things we call ‘ice cream headaches’ sometimes, and it’s just like…it kind of just feels like an ice cream headache… (ID 10)*
Mood swings	*I’m much more quickly to anger. I don’t handle stress as well. I’m very overwhelmed during that time, and it just steadily gets worse, whether or not it’s from pain or from the changes with my hormone levels, I don’t know, but it’s just a thing that happens. (ID 14) *
	*Like, I would have really bad mood swings and become really over dramatic [inaudible] emotional. (ID 8)*
	*My moods…when I notice my moods are changing or somethings…I can tell that I’m getting off, that means that my growth hormone is usually going up. (ID 15)*

*“Joint pain, but my doctor, when I was little, thought that maybe it might’ve been rheumatoid arthritis, and then osteoarthritis. And now, we’re kind of leaning towards it just being side effects of the acromegaly, because I do notice that when I take my shot, for 3 weeks, I’m okay. And then about the last week, I start having breakthrough symptoms where I start the swelling again, and my joints hurt…” (ID 13)*

*“The swelling in my face doesn’t happen for, you know, like a month or so after I’ve missed my shot completely, and I’m not just late or whatever. So, the swelling, the joint pain gets really bad, and then my mood is affected too. Within a couple of days. I mean, the…especially the joint pain kind of tapers, ah, to, you know, that more bearable level within like 3 days of the injection. It doesn’t take long at all, in the span of…you know, or in the…in the site of 28 days total. The swelling goes down probably right around day 2, to where that bearable stage is. So, I mean, it doesn’t take long at all after the injection to kind of start helping with those breakthrough symptoms.” (ID 14)*

*“You know, I start to have a difficult time processing emotions right before I get my injections. It’s in the week before it’s due. I didn’t recognize the pattern even though a lot of my friends had mentioned it before. But, actually, my daughter pointed it out most recently. [laughter] She’s like ‘I know, Mom. I know. It’s time for your shot.‘” (ID 1)*

The cognitive debriefing portion indicated that participants were able to interpret and understand the ASD items and had no issues with the 24-hour recall period.

*“How I felt in the last 24 hours.” (ID 11)*

*“To let them know the…if you’ve had any issues, or if you had, the severity of that particular issue within the last day.” (ID 14)*

**Fig. 1:**
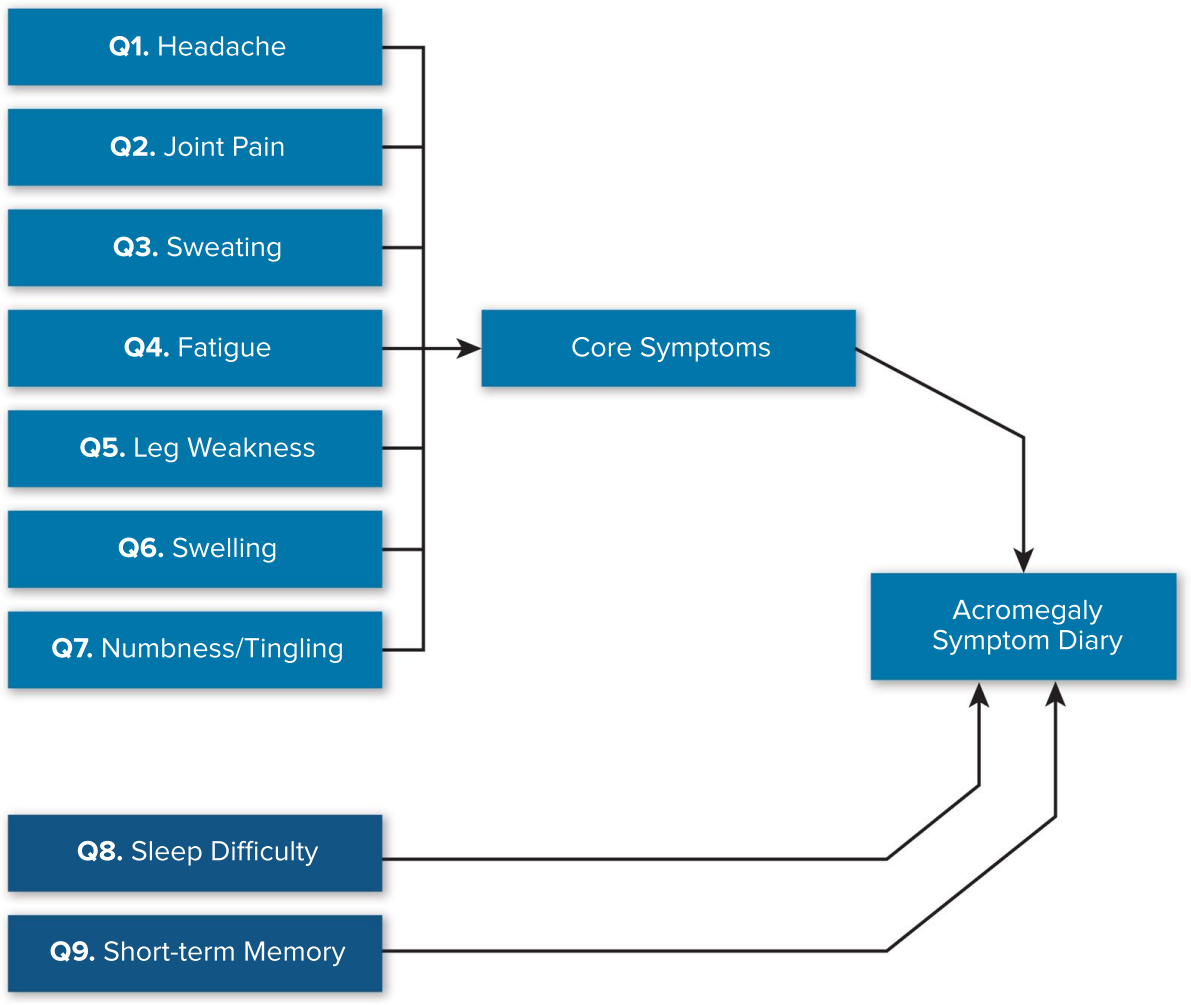
ASD conceptual framework ASD = Acromegaly Symptom Diary

Four participants reported having short-term memory difficulty as a symptom; therefore, a new item (Item 9) was added to include this concept in the ASD conceptual framework (Fig. 1).

*“I am very, very forgetful. That is actually one of the symptoms. I don’t know if it’s the tumor itself or if the surgery, but I have very bad short-term memory.“ (ID 3)*

*“Mental confusion. We call it the acro fog. I have, I have short-term memory deficiencies. I have cognitive issues.” (ID 5)*

*“I have memory loss due to this as well. I thought it was age. But my cousins are all the same age as me, and they’re not having any memory loss.” (ID 7)*

*“I have left my groceries at the store. I mean, I’ve left behind in the past month, twice. I had to go back and retrieve from the store. I’ve forgotten my doctor’s appointment time. Like, and then I forgot the time of this one today. Even though I had it checked, I’ve looked at the email, I still got it mixed up and didn’t comprehend that the interview was today. I thought it was yesterday, and I was emailing trying to get it straight.” (ID 8)*

When asked to identify the most important item on the ASD (not necessarily the most experienced symptom), the Fatigue item was most frequently reported, followed by Joint Pain (Figure S-1, Additional file 1).

“*My fatigue right now is a 7. It’s better than most days, but it’s still there. It doesn’t go away.“ (ID 1)*

*“But probably I would say 4 or 5. Maybe close to a 5. I would probably be very stationary and not have a lot of motivation to do anything.” (ID 2)*

*“Probably just a 4 because I kept falling asleep on the couch yesterday. It is very hard to get up in the morning, but I am able to eventually get up. I do eventually go to bed at night and fall asleep and sleep through the night. So I can’t say it’s the worst possible fatigue.” (ID 3)*

*“I’m sorry. I would say my joint pain comes and goes in my left hip at times when I bend over. When it does happen, it’s about a 4.” (ID 8)*

*“I kind of went a little bit in between 7 and 8 because also yesterday morning hours I had …my joints always bother me, but my left leg is standing out in that pain because it was just like every step I took, it just felt like things were just stretching in that leg.” (ID 10)*

*“Well, since I had joint pain last night, so I would give it a 6.” (ID 11)*

*“So, I would probably say, like, a 3. Usually the only time I feel my joint pain is when I walk up and down the stairs. Occasionally, I’ll be walking down the stairs, and I’ll be holding my son, and I feel just, like, my knee is hurting and my foot is hurting. But I kind of just…I don’t stop, I just work through it and then it goes away. So, it’s not, it’s not like an unbearable pain for me at this point.” (ID 12)*

### Psychometric evaluation

#### Demographics and characteristics

Participants in ACROBAT Evolve (N = 13) were mostly male (53.8%) and European (53.8%) and had a median age of 50.0 years. Participants in ACROBAT Edge (N = 47) were mostly female (57.4%), White (89.4%), and European (51.1%) and had a median age of 51.0 years. The mean duration since diagnosis was 144.7 months in ACROBAT Evolve and 113.3 months in ACROBAT Edge. The mean IGF-I was 0.8 × ULN, and mean GH was 0.9 ng/mL in ACROBAT Evolve. The mean IGF-I was 1.2 × ULN and mean GH was 1.9 ng/mL in ACROBAT Edge (Table 3). Descriptive statistics for key supporting measures and response frequencies for the patient-reported global items used in the evaluation are presented in Tables S-3 and S-4, respectively (Additional file 1).

**Table 3:** Characteristics of patients across both ASD clinical trials

Patient characteristics	ACROBAT Evolve (N = 13)	ACROBAT Edge (N = 47)
Sex, n (%)		
Female	6 (46.2)	27 (57.4)
Male	7 (53.8)	20 (42.6)
Age, years		
Mean ± SD, median, n	53.5 ± 13.76, 50.0, 13	52.0 ± 11.08, 51.0, 47
Min, Max	29.0, 75.0	31.0, 71.0
Country, n (%)		
North America	2 (15.4)	8 (17.0)
Europe	7 (53.8)	24 (51.1)
Rest of world	4 (30.8)	15 (31.9)
**Acromegaly disease characteristics**		
Duration since diagnosis, months		
Mean ± SD, median, n	144.7 ± 89.39, 145.3, 13	113.3 ± 69.16, 112.5, 47
Min, Max	17.2, 253.1	23.2, 365.1
Tumor size prior to pituitary surgery, mm		
Mean ± SD, median, n	15.8 ± 6.82, 15.0, 9	18.6 ± 9.6, 17.0, 42
Min, Max	6.0, 26.0	1.0, 43.0
Prior pituitary surgery (Yes), n (%)	13 (100.0)	37 (78.7)
IGF-I postoperative (× nmol/L)		
Mean ± SD, median, n	65.7 ± 27.13, 58.6, 13	71.4 ± 28.0, 60.8, 37
Min, Max	34.4, 117.6	33.0, 139.6
GH (ng/mL)		
Mean ± SD, median, n	0.9 ± 0.88, 0.6, 13	1.9 ± 3.7, 0.9, 47
Min, Max	0.1, 3.4	0.1, 23.9

ASD = Acromegaly Symptom Diary; GH = growth hormone; IGF-I = insulin-like growth factor I; SD = standard deviation; ULN = upper limit of normal

#### ASD item and composite level

Daily completion rates of the ASD (Items 1–7) were generally high across the 7 total days of the Baseline and Follow-up periods, with 96.7% (30/31) of participants completing at least 6 days during Baseline and 96.3% (26/27) completing at least 6 days during Follow-up Week 17. Most participants had scores for each item in the lower range (< 2 points) where Daily Joint Pain (Item 2) scores tended to have the largest averages (2.25-3 points), while Numbness/Tingling (Item 7) tended to have the lowest (1.2–1.6 points).

The item-level ASD scores were averaged across days to generate an ASD total score for the Screening Visit, Baseline, EOT, and Follow-up Week 17 timepoints (Table 4). The ASD total score at Screening Visit was 14.7 (SD = 13.7; range, 3.5–22.9) on the 0 to 70 scale. With 2.5% of patients having a score of 0, the ceiling effect was minimal for the total score at Screening Visit. The scores generally declined, on average, over both the screening and treatment periods. Mean change scores were − 1.2 from Screening Visit to Baseline and − 2.4 from Baseline to EOT, indicating small levels of improvement. Without treatment, the mean change was + 2.1 from EOT (Week 13) to end of wash-out (Week 17), which was a similar gain in magnitude to the loss from screening. No floor effect was identified at Screening or Baseline, indicating considerable potential for worsening. These findings are consistent with enrollment criteria requiring all patients to be on stable acromegaly treatment regimens. The ceiling effects observed at Screening and Baseline for five of the seven ASD items comprising the ASD total score (i.e., Headache Pain, Sweating, Leg Weakness, Swelling, and Numbness/Tingling) were consistent with the study enrollment criteria, based on IGF-I and GH values. As expected, ceiling effects increased across all items by EOT (potentially indicative of treatment effect) then decreased at Follow-up Week 17 (i.e., without treatment). The ASD total score was capable of demonstrating worsening in the overall response to treatment withdrawal from EOT to Follow-up Week 17. The observed ceiling and floor effects are not considered an indication of problematic ASD measurement quality.

**Table 4:** ASD Total Score, Item 8, and Item 9 across study timepoints

Timepoint	n	Mean ± SD	Q1, Median, Q3	Observed Min, Max	Score Min %, Max %
**ASD total score**					
Screening Visit	40	14.7 ± 13.7	3.5, 10.4, 22.9	0.0, 49.4	2.5, 0.0
Baseline	31	13.0 ± 12.5	3.4, 8.6, 22.0	0.0, 44.5	6.5, 0.0
EOT	42	10.9 ± 10.2	3.6, 7.8, 18.3	0.0, 40.1	11.9, 0.0
Week 17	27	12.6 ± 8.2	5.4, 13.1, 19.6	0.0, 30.7	7.4, 0.0
Change from screening visit to baseline	29	−1.2 ± 5.1	− 2.9, − 1.1, 0.7	−15.9, 16.7	NA
Change from baseline to EOT	27	−2.4 ± 7.0	−5.0, 0.0, 2.1	−24.7, 5.3	NA
Change from EOT to Week 17	25	2.1 ± 5.2	−0.3, 1.6, 5.9	−8.3, 12.4	NA
**Sleep difficulties (Item 8)**					
Screening visit	40	2.5 ± 2.6	0.2, 1.6, 3.9	0.0, 9.6	22.5, 0.0
Baseline	31	2.2 ± 2.7	0.3, 1.3, 3.6	0.0, 10.0	19.4, 3.2
EOT	42	2.1 ± 2.5	0.0, 1.4, 3.4	0.0, 9.0	33.3, 0.0
Week 17	27	2.0 ± 2.4	0.0, 1.2, 3.2	0.0, 9.0	25.9, 0.0
Change from screening visit to baseline	29	− 0.4 ± 0.9	− 0.7, -0.3, 0.0	− 2.5, 1.4	NA
Change from baseline to EOT	27	0.0 ± 2.4	− 0.7, 0.1, 0.7	− 5.4, 6.9	NA
Change from EOT to Week 17	25	− 0.2 ± 1.5	− 0.1, 0.0, 0.4	− 5.9, 2.1	NA
**Short-term memory (Item 9)**					
Screening visit	29	2.1 ± 2.2	0.3, 1.4, 3.1	0.0, 8.7	17.2, 0.0
Baseline	20	2.3 ± 2.6	0.1, 1.4, 3.7	0.0, 8.5	25.0, 0.0
EOT	33	1.5 ± 2.0	0.0, 1.0, 2.0	0.0, 7.7	36.4, 0.0
Week 17	17	2.2 ± 2.2	0.8, 1.9, 2.7	0.0, 9.0	23.5, 0.0
Change from screening visit to baseline	19	0.1 ± 1.2	− 0.4, 0.0, 0.3	− 1.6, 2.9	NA
Change from baseline to EOT	16	− 0.4 ± 1.3	− 0.5, 0.0, 0.2	− 4.6, 0.9	NA
Change from EOT to Week 17	15	0.0 ± 1.5	− 0.3, 0.0, 0.8	− 4.0, 3.0	NA

ASD = Acromegaly Symptom Diary; EOT = end of treatment; NA = not assessed; Q1 = quartile 1 (25th percentile); Q3 = quartile 3 (75th percentile); SD = standard deviation

The ASD total score ranges from 0 to 70.

Across the data from both clinical trials, strong (r ≥ 0.50) IICs were found with ASD Items 1–7 at Baseline, where each item was correlated with at least 3 to 4 other items, and no items showed a correlation lower than 0.3 (Table 5). The two strongest IICs across Items 1–7 at Baseline were 0.82 for the correlation between Headache Pain (Item 1) and Fatigue (Item 4) and 0.83 for the correlation between Joint Pain (Item 2) and Numbness/Tingling (Item 7). The smallest correlation was 0.37 for the correlation between Swelling (Item 6) and Sweating (Item 3). At Week 17, the IICs remained moderate to strong across Items 1–7, but the overall IIC strength was smaller than at Baseline (all magnitudes across Items 1–7 < 0.80 but ≥ 0.10. Cronbach’s coefficient alpha was 0.91 at Baseline for ASD items, and 0.80 at Week 17, suggesting good measurement consistency within the items and supporting an ASD total score [11, 22].

**Table 5:** Inter-item correlations between ASD item-level scores and alpha scores for internal consistency

Timepoint/ASD Item	Inter-item Correlation								
	1	2	3	4	5	6	7	8	9
**Baseline (n = 20–31)**									
Item 1. Headache pain	NA	﻿NA	﻿NA	﻿NA	﻿NA	﻿NA	﻿NA	﻿NA	﻿NA
Item 2. Joint pain	0.62	﻿NA	﻿NA	﻿NA	﻿NA	﻿NA	﻿NA	﻿NA	﻿NA
Item 3. Sweating	0.42	0.73	﻿NA	﻿NA	﻿NA	﻿NA	﻿NA	﻿NA	﻿NA
Item 4. Fatigue	0.82	0.77	0.55	﻿NA	﻿NA	﻿NA	﻿NA	﻿NA	﻿NA
Item 5. Leg weakness	0.59	0.68	0.40	0.70	﻿NA	﻿NA	﻿NA	﻿NA	﻿NA
Item 6. Swelling	0.54	0.72	0.37	0.52	0.59	﻿NA	﻿NA	﻿NA	﻿NA
Item 7. Numbness/tingling	0.52	0.83	0.46	0.69	0.56	0.64	﻿NA	﻿NA	﻿NA
Item 8. Sleep difficulty	0.67	0.72	0.65	0.79	0.69	0.44	0.61	﻿NA	﻿NA
Item 9. Short-Term Memory	0.59	0.76	0.55	0.71	0.89	0.68	0.69	0.69	﻿NA
Coefficient alpha without column item	0.93	0.92	0.93	0.93	0.92	0.94	0.93	0.93	0.92
Coefficient alpha (all items)	0.94	﻿NA	﻿NA	﻿NA	﻿NA	﻿NA	﻿NA	﻿NA	﻿NA
Coefficient alpha without column item (only Items 1–7)	0.90	0.87	0.92	0.88	0.89	0.90	0.89	﻿NA	﻿NA
Coefficient alpha (only Items 1–7)	0.91	﻿NA	﻿NA	﻿NA	﻿NA	﻿NA	﻿NA	﻿NA	﻿NA
**Week 17 (n = 17–27)**									
Item 1. Headache pain	﻿NA	﻿NA	﻿NA	﻿NA	﻿NA	﻿NA	﻿NA	﻿NA	﻿NA
Item 2. Joint pain	0.16	﻿NA	﻿NA	﻿NA	﻿NA	﻿NA	﻿NA	﻿NA	﻿NA
Item 3. Sweating	0.31	0.37	﻿NA	﻿NA	﻿NA	﻿NA	﻿NA	﻿NA	﻿NA
Item 4. Fatigue	0.55	0.36	0.34	﻿NA	﻿NA	﻿NA	﻿NA	﻿NA	﻿NA
Item 5. Leg weakness	0.35	0.56	0.18	0.72	﻿NA	﻿NA	﻿NA	﻿NA	﻿NA
Item 6. Swelling	0.17	0.58	0.28	0.17	0.23	﻿NA	﻿NA	﻿NA	﻿NA
Item 7. Numbness/tingling	−0.10	0.62	0.49	0.47	0.38	0.57	﻿NA	﻿NA	﻿NA
Item 8. Sleep difficulty	0.18	0.63	0.47	0.55	0.70	0.28	0.53	﻿NA	﻿NA
Item 9. Short-term memory	−0.26	0.45	0.17	0.31	0.56	0.59	0.74	0.69	﻿NA
Coefficient alpha without column item	0.88	0.83	0.86	0.84	0.80	0.85	0.83	0.79	0.83
Coefficient alpha (all items)	0.85	﻿NA	﻿NA	﻿NA	﻿NA	﻿NA	﻿NA	﻿NA	﻿NA
Coefficient alpha without column item (only Items 1–7)	0.81	0.75	0.79	0.76	0.76	0.79	0.77	﻿NA	﻿NA
Coefficient alpha (only Items 1–7)	0.80	﻿NA	﻿NA	﻿NA	﻿NA	﻿NA	﻿NA	﻿NA	﻿NA

ASD = Acromegaly Symptoms Diary; NA = ﻿not assessed

#### Test-retest reliability

The ASD total score ICC was 0.90 (95% confidence interval [CI], 0.80–0.95), and the item-level ICCs comprising the total score ranged from 0.74 for Leg Weakness (Item 5) to 0.98 for Sweating (Item 3). Sleep Difficulty (Item 8) and Short-Term Memory (Item 9) had ICCs of 0.88 and 0.86, respectively. All ICCs exceeded the recommended threshold of 0.70, indicating ASD total scores were stable over time relative to the heterogeneity of the studied patients [25].

#### Validity

Strong correlations were observed at Baseline between the ASD total scores and the PGI-S (r = 0.65), and the correlations between the ASD items and the PGI-S were moderate to strong, ranging from 0.38 to 0.62 at Baseline (Table 6). The correlations between the ASD total score and the two general health-related QOL scores (the AcroQoL total score and EQ-VAS) were strong (r = − 0.61 and − 0.53, respectively). At Week 17, this pattern continued, with strong correlations between the ASD total score and the PGI-S (r = 0.67) and moderate to strong correlations with the AcroQoL total score (r = -0.66) and EQ-VAS (r = − 0.47). Overall, relationships in the expected directions between the total ASD score and supporting measures were confirmed. Correlations observed at Week 17 between Sleep Difficulty (Item 8) and the PGI-S (r = 0.27), the AcroQoL total score (r = − 0.38) and EQ-VAS (r = − 0.36) were weaker. A similar pattern was seen for correlations at Week 17 between Short-Term Memory (Item 9) and the PGI-S (r = 0.25), the AcroQoL total score (r = − 0.31) and EQ-VAS (r = − 0.31).

**Table 6:** Construct validity correlations of ASD total scores

ASD score	Correlations with supporting measures		
	PGI-S	EQ-VAS	AcroQoL Total
**Baseline (n = 19 to 31)**			
Total score	0.65*	− 0.53*	− 0.61*
Item 1. Headache pain	0.38*	− 0.41*	− 0.44*
Item 2. Joint pain	0.58*	− 0.60*	− 0.52*
Item 3. Sweating	0.52*	− 0.36*	− 0.40*
Item 4. Fatigue	0.62*	− 0.40*	− 0.57*
Item 5. Leg weakness	0.53*	− 0.42*	− 0.52*
Item 6. Swelling	0.59*	− 0.41*	− 0.54*
Item 7. Numbness/tingling	0.43*	− 0.42*	− 0.47*
Item 8. Sleep difficulty	0.42*	− 0.37*	− 0.40*
Item 9. Short-term memory	0.56*	− 0.51*	− 0.53*
**Week 17 (n = 16 to 27)**			
Total Score	0.67*	− 0.47*	− 0.66*
Item 1. Headache pain	0.54*	− 0.27	− 0.43*
Item 2. Joint pain	0.50*	− 0.35	− 0.45*
Item 3. Sweating	**0.19**	− 0.15	− 0.25
Item 4. Fatigue	0.49*	− 0.37	− 0.58*
Item 5. Leg weakness	0.43*	− 0.53*	− 0.47*
Item 6. Swelling	0.68*	− 0.30	− 0.44*
Item 7. Numbness/‌tingling	**0.37**	− 0.17	− 0.46*
Item 8. Sleep difficulty	**0.27**	− 0.36	− 0.38
Item 9. Short-term memory	**0.25**	− 0.31	− 0.31

*P < 0.05 test for H0: ρ = 0

AcroQoL = Acromegaly Quality of Life Questionnaire; ASD = Acromegaly Symptoms Diary; EQ-VAS = EQ visual analogue scale; PGI-S = Patient Global Impression of Severity

Bold values indicate correlation values were hypothesized to be moderate to strong

Patients with PGI-S ratings “none” or “mild” had significantly (*P* < 0.01) lower (better) mean ± SD ASD total scores (Baseline 7.76 ± 8.1; Week 17: 8.98 ± 7.4) than patients with PGI-S ratings of “moderate” or “severe” (Baseline: 23.95 ± 13.4; Week 17: 17.05 ± 7.1). At Baseline, the mean difference was statistically significant (*P* < 0.05) for all items (except Short-Term Memory), and at Week 17, the difference was significant (*P* < 0.05) for Headache Pain (Item 1), Joint Pain (Item 2), and Swelling (Item 6). A statistical test was not conducted because of small sample sizes, but a trend in the ASD total score was observed at Week 17, as patients with IGF-I ≤ 1 × ULN had a lower mean ASD total score (mean = 11.75, SD = 13.2) than patients with IGF-I > 2.5 × ULN (mean = 15.84 ± 6.3).

#### Ability to detect change

Change in ASD scores was anticipated during Screening Visit to Baseline and EOT to Week 17. The largest average ASD total deterioration (increase in scores) was 5.49 (SD = 4.8) in the subgroup of patients with worsened scores on the PGI-S from EOT to Week 17 (n = 8, *P* < 0.05). The responsiveness correlations were trivial to small between changes in ASD total score and changes in PGI-S, IGF-I, and GH from Screening Visit to Baseline (|r| ≤ 0.19) because of the restricted range in the distribution of change scores during this period (Table 7). However, during the time between EOT and Week 17, all correlations were strong between ASD total change scores and changes in PGI-S (r = 0.53), IGF-I (r = 0.52), and GH (r = 0.56), suggesting good ability to detect change within the ASD. The magnitude of the correlation (0.50 < r ≤ 0.56) indicates that the ASD total change scores provide complementary, yet unique, information regarding change in disease status relative to the change in the biomarkers IGF-I and GH.

**Table 7:** Responsiveness correlations of the ASD weekly total scores

Change/supporting measure	Correlation with change in ASD Total
Change from screening visit to baseline (n = 29)	
PGI-S	0.19
IGF-I (× ULN)	− 0.03
Growth hormone (ng/mL)	0.06
Change from EOT to Week 17 (n = 24 to 25)	
PGI-S	0.53*
IGF-I (× ULN)	0.52*
Growth hormone (ng/mL)	0.56*
AcroQoL total score	− 0.08
EQ-5D-5 L pain/discomfort	− 0.06
EQ-5D-5 L mobility	− 0.11
EQ-5D-5 L self-care	− 0.01
EQ-5D-5 L usual activities	− 0.12
EQ-5D-5 L anxiety/depression	− 0.21
EQ-VAS	− 0.08

*P < 0.05 test for H0: ρ = 0

ASD = Acromegaly Symptom Diary; EOT = end of treatment; EQ-VAS = EQ visual analogue scale; IGF-I = insulin-like growth factor I; ULN = upper limit of normal; PGI-S = Patient Global Impression of Severity

#### Meaningful within-patient change

The pattern of ASD total change scores from EOT to Week 17 across different change levels of PGI-S was as anticipated, and the responsiveness correlation was 0.53. From EOT to Week 17, the mean ± SD change in the PGI-S in the 1-point improvement group (n = 2) was − 3.64 ± 5.76 (median = − 3.64), whereas the mean ± SD change in the 1-point worsening group (n = 7) was 5.05 ± 5.01 (median = 3.71). The 95% CI of the no-change group (n = 15) was − 1.37 to 3.61; therefore, despite small sample sizes in the improvement and worsening groups, the magnitude of the threshold based on the median change exceeded the absolute largest limit of the 95% CI of the no-change group. Considering this result, along with the distribution-based estimates (half SD = 6.3, SEM = 4.0) [30, 31, 33], the proposed preliminary threshold range to characterize a meaningful change from the patients’ perspective for the ASD total is a 4- to 6-point change for improvement or worsening (Table 8).

**Table 8:** Meaningful within-person thresholds of ASD total scores, Item 8, and Item 9

Change in PGI-S	n	Change in ASD total from EOT to Week 17			
		Mean ± SD	Q1, Median, Q3	Min, Max	95% CI
1-point improvement	2	− 3.64 ± 5.76	− 7.71, − 3.64, 0.43	− 7.71, 0.43	− 55.38 to 48.09
No difference	15	1.12 ± 4.50	− 1.12, 0.50, 4.21	− 8.29, 7.86	− 1.37 to 3.61
1-point worsening	7	5.05 ± 5.01	0.14, 3.71, 9.14	− 0.29, 12.43	0.42 to 9.68
2-point worsening	1	8.57 (−)	8.57, 8.57, 8.57	8.57, 8.57	NA
**Change in sleep difficulty (Item 8) From EOT to Week 17**					
3-point improvement	0	NA	NA	NA	NA
2-point improvement	0	NA	NA	NA	NA
1-point improvement	2	− 1.50 ± 1.92	− 2.86, − 1.50, − 0.14	− 2.86, − 0.14	− 18.74 to 15.74
No difference	15	− 0.39 ± 1.66	− 0.57, 0.00, 0.43	− 5.86, 1.57	− 1.30 to 0.53
1-point worsening	7	0.69 ± 0.97	0.00, 0.00, 1.95	0.00, 2.14	− 0.21 to 1.58
2-point worsening	1	0.00 (-)	0.00, 0.00, 0.00	0.00, 0.00	NA
3-point worsening	0	NA	NA	NA	NA
**Change in Short-Term Memory (Item 9) From EOT to Week 17**					
3-point improvement	0	NA	NA	NA	NA
2-point improvement	0	NA	NA	NA	NA
1-point improvement	1	− 2.00 (-)	− 2.00, − 2.00, − 2.00	− 2.00, − 2.00	NA
No difference	10	0.19 ± 1.80	− 0.14, 0.20, 1.23	− 4.00, 3.00	− 1.10 to 1.47
1-point worsening	4	− 0.01 ± 0.21	− 0.14, 0.00, 0.12	− 0.29, 0.24	− 0.35 to 0.33
2-point worsening	0	NA	NA	NA	NA
3-point worsening	0	NA	NA	NA	NA

ASD = Acromegaly Symptom Diary; CI = confidence interval; EOT = end of treatment; max = maximum; min = minimum; NA = not assessed; PGI-S = Patient Global Impression of Severity; Q1 = quartile 1 (25th percentile); Q3 = quartile 3 (75th percentile)

Groups with an n of 0 were omitted from the table.

## Discussion

The ASD is a daily PRO instrument designed for detecting changes in symptoms of acromegaly in adult participants during clinical trials and was developed in a manner consistent with FDA guidance. The results from the concept elicitation interviews identified the most important aspects of acromegaly symptoms for use in developing the conceptual framework of the ASD. The cognitive debriefing interviews showed that the ASD items were relevant to the experiences of individuals with acromegaly and that the ASD items were correctly interpreted, easily completed, and recalled (within 24 h) by the participants. The initial psychometric evaluation of the measurement properties, using the available data collected in two phase 2 clinical studies [16, 17], demonstrated that the ASD can measure acromegaly symptom severity in a valid and reliable manner. Additionally, the psychometric evaluation provided evidence to support the computation of the ASD total score for symptom monitoring and supported the ASD total score as responsive to change. The ASD total score showed acceptable distributional item-level characteristics, measurement structure, internal consistency, test-retest reliability, construct validity (convergent and divergent validity, known-groups validity), and ability to detect change. Moderate to strong correlations were found between the ASD and supporting measures (PGI-S, AcroQoL, and EQ-VAS). A preliminary threshold range of a 4- to 6-point reduction is proposed to characterize meaningful within-patient change in the ASD total score.

The ASD shares similarities in core symptom (physical and psychological) content with current PRO measures such as the AcroQoL and the Acro-TSQ, which have both been used in clinical and research settings [8, 11, 14, 15]. Although the Acro-TSQ is designed to assess treatment-related effects specifically, it can also assess the impact of acromegaly symptoms on health-related QOL [8]. One primary feature that distinguishes the ASD from other acromegaly PROs is the 24-hour recall period for reporting symptoms. In contrast, the AcroQoL has a broader unspecified recall period and the Acro-TSQ has a variable recall period depending on the treatment regimen (Acro-TSQ asks about symptom interference since the last injection) [8, 11]. While the measurement properties of the AcroQoL and the Acro-TSQ have been well supported by previous psychometric evaluation research [8, 11, 14, 15], the more immediate, day-to-day changes in symptoms should be considered, and the recall periods of these two measures are not aligned with FDA guidance [9, 10]. The 24-hour recall period of the ASD is aligned with this guidance that PRO measures involving memory recall have a specified recall period that occurs over a short time period. This necessary alignment with FDA guidance meets the context of use criteria for the ASD to be used in future acromegaly clinical trials.

Given that acromegaly is characterized by increases in GH and IGF-I, there is a need to monitor hormone levels [34, 35], and a disease-specific PRO measure like the ASD that captures daily symptom changes could enhance clinical assessments. Although the PASQ [12], a disease-specific questionnaire that evaluates five symptoms and signs of acromegaly (soft-tissue swelling, arthralgia, headache, excessive perspiration, and fatigue), has been widely used, this PRO measure has not been validated [13]. Indeed, most PRO measures used in trials for acromegaly have not been validated, and this lack of validation prevents accurate comparison of outcomes across trials and implementation of findings into clinical practice [13].

This study has limitations that should be considered. Although the sample sizes in the clinical trials were small, pooling both samples provided sufficient evidence for examining measurement properties. Another limitation was that majority of the sample population for the concept elicitation and cognitive debriefing interviews were female (68.8%) and White (93.8%), which is a higher proportion than the approximately evenly distributed prevalence of acromegaly among males and females [36]. The disproportionate race and sex composition of the sample may limit the representativeness of the study findings. Additionally, the ACROBAT studies were designed to maintain biochemical control achieved with injected SRLs after switching injected therapy to oral paltusotine and thus may have contributed to the observed ceiling effects at baseline and during treatment. This may have made meaningful change (improvement or worsening) in symptom detection on the ASD more difficult. However, the ASD total score was shown to be capable of demonstrating worsening during treatment withdrawal.

Importantly, Items 8 and 9 (Sleep Difficulty and Short-Term Memory) were not included in the computation of the total score following a recommendation from the FDA. As the context of use of the ASD is in clinical trials evaluating treatments for acromegaly, we recommend that Items 8 and 9 not be included in the overall score when used in such trials. Even though these two items do not contribute to the overall score of the ASD, these items were part of the development of the ASD and were psychometrically evaluated. Furthermore, these concepts were important to participants and should be monitored. As such, we have opted to still present individual data for Items 8 and 9 despite these items not being included in the ASD overall score. Finally, it is unknown if these results are generalizable to treatment-naïve patients with acromegaly because all participants in the ACROBAT trials were treated with stable doses of pharmacological treatment and were relatively asymptomatic with only mild elevation of IGF-I levels at study entry.

## Conclusion

These findings provide qualitative and quantitative evidence to support the ASD as fit for the purpose of evaluating the symptom experience of patients with acromegaly in clinical trials.

## Supplementary Information

**Additional file 1.** Supplementary Materials.

## Data Availability

Qualitative data are primarily in the form of transcripts and cannot be made available to protect participant privacy in accordance with the principles of the Belmont Report.

## Abbreviations

AcroQoL: Acromegaly Quality of Life Questionnaire
Acro-TSQ: Acromegaly Treatment Satisfaction Questionnaire
ANOVA: Analysis of variance
ASD: Acromegaly Symptom Diary
CI: confidence interval
EOT: end of treatment
EQ-VAS: EQ visual analog scale
FDA: US Food and Drug Administration
GED: General Educational Development
GH: growth hormone
ICC: intraclass correlation coefficient
IGF-I: insulin-like growth factor I
IIC: inter-item correlation
LAR: long-acting release
PGI-I: Patient Global Impression of Improvement
PGI-S: Patient Global Impression of Severity
PRO: patient-reported outcome
Q1: quartile 1
Q3: quartile 3
QOL: quality of life
SD: standard deviation
SEM: standard error of the measurement
SRL: somatostatin receptor ligand
ULN: upper limit of normal
US: United States

## References

[1] Melmed S (2009). Acromegaly pathogenesis and treatment. J Clin Invest.

[2] Colao A, Ferone D, Marzullo P (2004). Systemic complications of acromegaly: epidemiology, pathogenesis, and management. Endocr Rev.

[3] Mercado M, Ramirez-Renteria C (2018). Metabolic complications of acromegaly. Front Horm Res.

[4] Sharma MD, Nguyen AV, Brown S (2017). Cardiovascular disease in acromegaly. Methodist Debakey Cardiovasc J.

[5] Hannah-Shmouni F, Trivellin G, Stratakis CA (2016). Genetics of gigantism and acromegaly. Growth Horm IGF Res.

[6] Abreu A, Tovar AP, Castellanos R (2016). Challenges in the diagnosis and management of acromegaly: a focus on comorbidities. Pituitary.

[7] Strasburger CJ, Karavitaki N, Stormann S (2016). Patient-reported outcomes of parenteral somatostatin analogue injections in 195 patients with acromegaly. Eur J Endocrinol.

[8] Fleseriu M, Fogelfeld L, Gordon MB (2020). An evaluation of the Acromegaly treatment satisfaction questionnaire (Acro-TSQ) in adult patients with acromegaly, including correlations with other patient-reported outcome measures: data from two large multicenter international studies. Pituitary.

[9] US Food and Drug Administration (2009) Patient-reported outcome measures: use in medical product development to support labeling claims. https://www.fda.gov/regulatory-information/search-fda-guidance-documents/patient-reported-outcome-measures-use-medical-product-development-support-labeling-claims 10.1186/1477-7525-4-79 PMC1629006 17034633

[10] US Food and Drug Administration. Patient-focused drug development guidance: methods to identify what is important to patients and select, develop or modify fit-for-purpose clinical outcome assessments (2018) https://www.fda.gov/drugs/news-events-human-drugs/patient-focused-drug-development-guidance-methods-identify-what-important-patients-and-select

[11] Badia X, Webb SM, Prieto L (2004). Acromegaly Quality of Life Questionnaire (AcroQoL). Health Qual Life Outcomes.

[12] Trainer PJ, Drake WM, Katznelson L (2000). Treatment of acromegaly with the growth hormone-receptor antagonist pegvisomant. N Engl J Med.

[13] van der Meulen M, Zamanipoor Najafabadi AH, Broersen LHA (2021). State of the art of patient-reported outcomes in acromegaly or GH deficiency: a systematic review and meta-analysis. J Clin Endocrinol Metab.

[14] Fleseriu M, Molitch M, Dreval A (2021). Disease and treatment-related burden in patients with acromegaly who are biochemically controlled on injectable somatostatin receptor ligands. Front Endocrinol (Lausanne).

[15] Rowles SV, Prieto L, Badia X (2005). Quality of life (QOL) in patients with acromegaly is severely impaired: use of a novel measure of QOL: acromegaly quality of life questionnaire. J Clin Endocrinol Metab.

[16] ClinicalTrials.gov (2021) A study to evaluate the safety and efficacy of paltusotine for the treatment of acromegaly (ACROBAT Evolve). https://clinicaltrials.gov/ct2/show/NCT03792555

[17] ClinicalTrials.gov (2021) An study to evaluate the safety and efficacy of paltusotine for the treatment of acromegaly (ACROBAT edge). https://clinicaltrials.gov/ct2/show/NCT03789656

[18] Gadelha MR, Gordon MB, Doknic M (2022). ACROBAT Edge:&nbsp;&nbsp; Safety and efficacy of switching injected SRLs to oral paltusotine in patients with acromegaly. J Clin Endocrinol Metab.

[19] National Institute of Mental Health. Patient Global Impressions scale - Change, Improvement, Severity (PGI-C, PGI-I, PGI-S) (2021) https://eprovide.mapi-trust.org/instruments/patient-global-impressions-scale-change-improvement-severity

[20] EuroQol Research Foundation. About the EQ-5D (2021) https://euroqol.org/eq-5d-instruments/

[21] US Food and Drug Administration (2018) Methods to identify what is important to patients & select, develop or modify fit-for-purpose clinical outcomes assessments. https://www.fda.gov/media/116281/download

[22] Streiner DL, Norman GR, Cairney J (2015) Health measurement scales: a practical guide to their development and use. Oxford University Press

[23] Cronbach LJ (1951). Coefficient alpha and the internal structure of tests. Psychometrika.

[24] McGraw KO, Wong SP (1996). Forming inferences about some intraclass correlation coefficients. Psychol Methods.

[25] Nunnally J (1994). Psychometric theory 3ed.

[26] Cohen J (1992). A power primer. Psychol Bull.

[27] Fayers PM, Hays RD (2014). Don’t middle your MIDs: regression to the mean shrinks estimates of minimally important differences. Qual Life Res.

[28] Hays RD, Farivar SS, Liu H (2005). Approaches and recommendations for estimating minimally important differences for health-related quality of life measures. COPD.

[29] Revicki D, Hays RD, Cella D (2008). Recommended methods for determining responsiveness and minimally important differences for patient-reported outcomes. J Clin Epidemiol.

[30] Norman GR, Sloan JA, Wyrwich KW (2003). Interpretation of changes in health-related quality of life: the remarkable universality of half a standard deviation. Med Care.

[31] Wyrwich KW, Tierney WM, Wolinsky FD (1999). Further evidence supporting an SEM-based criterion for identifying meaningful intra-individual changes in health-related quality of life. J Clin Epidemiol.

[32] McLeod LD, Coon CD, Martin SA (2014). Interpreting patient-reported outcome results: US FDA guidance and emerging methods. Exp Rev Pharmacoecon Outcomes Res.

[33] Crosby RD, Kolotkin RL, Williams GR (2003). Defining clinically meaningful change in health-related quality of life. J Clin Epidemiol.

[34] Silverstein JM (2015). Need for improved monitoring in patients with acromegaly. Endocr Connect.

[35] Freda PU (2009). Monitoring of acromegaly: what should be performed when GH and IGF-1 levels are discrepant?. Clin Endocrinol (Oxf).

[36] Lavrentaki A, Paluzzi A, Wass JA (2017). Epidemiology of acromegaly: review of population studies. Pituitary.

