# Supplementary material for: Development and evaluation of the Acromegaly Symptom Diary
Source: J Patient Rep Outcomes. 2023 Feb 15;7:15. doi: 10.1186/s41687-023-00541-7 (PMC9931976; doi:10.1186/s41687-023-00541-7)
Supplement: Supplementary file 1 — Additional file 1. Supplementary Materials. [file 41687_2023_541_MOESM1_ESM.docx]

# Supplementary Material

Figure S-1. ASD Item 2 for Joint Pain


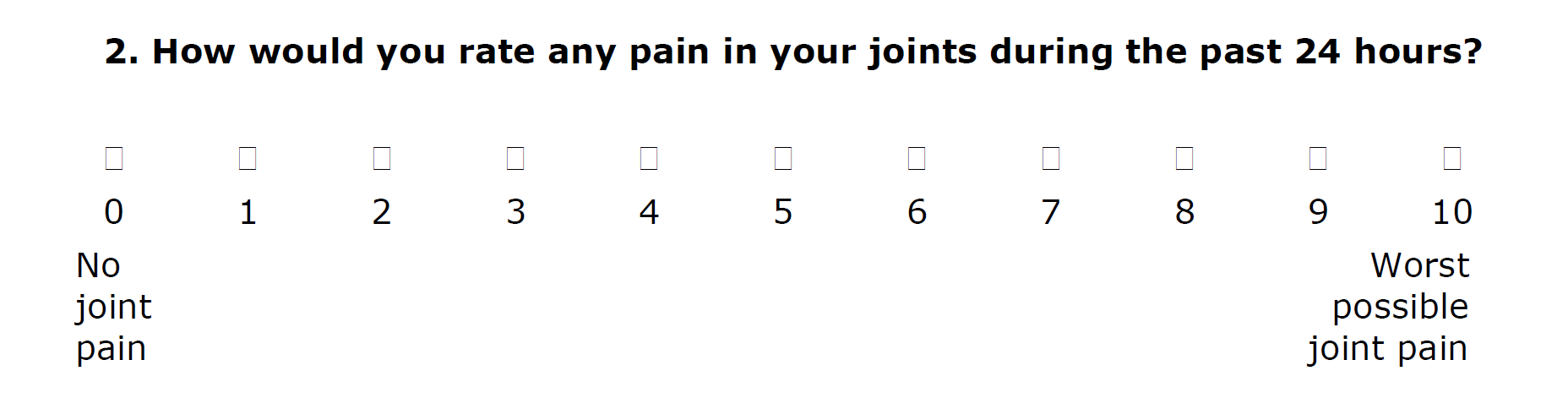


ASD = Acromegaly Symptom Diary.

Table S-1. Measures and Key Time Points Used in the ASD Psychometric Evaluation

| **Measure** | **Key Time Points Pooled Data From ACROBAT Evolve^1^ and Edge^2,3^ Trials** | |
| --- | --- | --- |
|  | **Screening** | **Follow-Up** |
| ASD | Visit 1b  Baseline (defined as the daily average for 7 days prior to Visit 3) | Daily average for 7 days prior to Week 17/Visit 16 |
| PGI-S | Visit 1b  Baseline (defined as the last value prior to first dose of study drug) | Week 17/Visit 16 (Day 119) |
| PGI-I | None | Week 17/Visit 16 (Day 119) |
| AcroQoL and EQ-5D | Baseline (defined as the last value prior to first dose of study drug) | Week 17/Visit 16 (Day 119) |
| IGF-I | Baseline (defined as the mean of Visits 1b, 2, and 3) | Week 17/Visit 16 (Day 119) |
| GH | Baseline (defined as the mean of Visits 1b and 2) | Week 17/Visit 16 (Day 119) |

AcroQoL = Acromegaly Quality of Life Questionnaire; ASD = Acromegaly Symptom Diary; GH = growth hormone; IGF-I = insulin-like growth factor I; PGI-I = Patient Global Impression of Improvement; PGI-S = Patient Global Impression of Severity.

'-' = no assessment.

Note: The Screening period is composed of Visits 1a, 1b, and 2.

^1^ClinicalTrials.gov. A study to evaluate the safety and efficacy of paltusotine for the treatment of acromegaly (ACROBAT Evolve). 2021. https://clinicaltrials.gov/ct2/show/NCT03792555.

^2^ClinicalTrials.gov. An study to evaluate the safety and efficacy of paltusotine for the treatment of acromegaly (ACROBAT Edge). 2021. https://clinicaltrials.gov/ct2/show/NCT03789656.

^3^Gadelha MR, Gordon MB, Doknic M, et al. (2022) ACROBAT Edge: Safety and efficacy of switching injected SRLs to oral paltusotine in patients with acromegaly. J Clin Endocrinol Metab. doi: 10.1210/clinem/dgac643.

Table S-2. Proposed *A Priori* Hypotheses

| ***A Priori* Hypotheses for Construct Validity** |
| --- |
| *Hypothesis 1:* Correlations are expected to be moderate to strong (\|r\| ≥ 0.3) and positive between the ASD item (and total) scores and the PGI-S, and EQ-5D-5L Pain/Discomfort item scores, supporting convergent validity. |
| *Hypothesis 2:* Correlations are expected to be smaller (\|r\| < 0.3) and negative between the ASD item (and total) scores and EQ-VAS and AcroQoL total scores due to the broader scope of these measures regarding the ASD, supporting divergent validity. |
| *Hypothesis 3:* Better (lower) ASD scores are expected on average for patient subgroups responding “none” or “mild” to the PGI-S than those responding “moderate” or “severe.” |
| *Hypothesis 4:* Better (lower) ASD scores are expected on average for patients with serum IGF-I within the normal range than those with serum IGF-I levels above the normal range (i.e., ≤ 1 x ULN and > 2.5 x ULN). |

AcroQoL = Acromegaly Quality of Life Questionnaire; ASD = Acromegaly Symptom Diary; IGF-I = insulin-like growth factor I; PGI-S = Patient Global Impression of Severity. ULN = upper limit of normal

Table S-3. Descriptive Statistics for Supporting Measures

| Time Point/Measure | n | Mean ± SD | Q1, Median, Q3 | Observed Min, Max | Score Min %, Max % |
| --- | --- | --- | --- | --- | --- |
| Screening Visit |  |  |  |  |  |
| GH (ng/mL) | 60 | 1.8 ± 3.4 | 0.4, 0.9, 1.7 | 0.1, 24.7 | - |
| IGF-I (× ULN) | 60 | 1.2 ± 0.5 | 0.9, 1.1, 1.5 | 0.5, 2.7 | - |
| Baseline |  |  |  |  |  |
| GH (ng/mL) | 60 | 1.7 ± 3.3 | 0.5, 0.8, 1.6 | 0.1, 23.9 | - |
| IGF-I (× ULN) | 60 | 1.1 ± 0.4 | 0.9, 1.0, 1.4 | 0.5, 2.3 | - |
| EQ-VAS | 47 | 73.6 ± 16.1 | 65.0, 75.0, 85.0 | 30.0, 100.0 | 0.0, 2.1 |
| AcroQoL total score | 59 | 76.7 ± 13.2 | 69.0, 75.0, 87.0 | 46.0, 102.0 | 0.0, 0.0 |
| EOT |  |  |  |  |  |
| GH (ng/mL) | 60 | 2.0 ± 4.6 | 0.5, 0.9, 2.0 | 0.1, 35.5 | - |
| IGF-I (× ULN) | 60 | 1.4 ± 0.6 | 1.0, 1.3, 1.6 | 0.6, 3.5 | - |
| EQ-VAS | 45 | 77.6 ± 14.7 | 70.0, 75.0, 90.0 | 40.0, 100.0 | 0.0, 8.9 |
| AcroQoL total score | 53 | 77.8 ± 14.9 | 67.0, 76.0, 91.0 | 48.0, 110.0 | 0.0, 1.9 |
| Week 17 |  |  |  |  |  |
| GH (ng/mL) | 58 | 3.2 ± 4.3 | 1.2, 1.9, 3.5 | 0.2, 29.4 | - |
| IGF-I (× ULN) | 58 | 2.0 ± 0.9 | 1.3, 1.8, 2.5 | 0.5, 4.8 | - |
| EQ-VAS | 44 | 76.4 ± 15.1 | 70.0, 75.0, 90.0 | 40.0, 98.0 | 0.0, 0.0 |
| AcroQoL total score | 57 | 76.6 ± 14.9 | 67.0, 75.0, 89.0 | 40.0, 110.0 | 0.0, 1.8 |

AcroQoL = Acromegaly Quality of Life Questionnaire; EOT = end of treatment at Week 13; EQ-VAS = EQ visual analogue scale; GH = growth hormone; IGF-I = insulin-like growth factor I; Q1 = quartile 1 (25th percentile); Q3 = quartile 3 (75th percentile).

Table S-4. Response Frequencies for the Patient-Reported Global Items

| **Study/Measure** | **Screening Visit^a^** | **Baseline** | **EOT** | **Week 17** |
| --- | --- | --- | --- | --- |
| **PGI-S** |  |  |  |  |
| **ACROBAT Evolve, n** | 12 | 12 | 12 | 11 |
| None (score = 0), n (%) | 4 (33.3) | 2 (16.7) | 2 (16.7) | 1 (9.1) |
| Mild (score = 1), n (%) | 5 (41.7) | 6 (50.0) | 7 (58.3) | 5 (45.5) |
| Moderate (score = 2), n (%) | 3 (25.0) | 4 (33.3) | 3 (25.0) | 5 (45.5) |
| Severe (score = 3), n (%) | 0 (0.0) | 0 (0.0) | 0 (0.0) | 0 (0.0) |
| **ACROBAT Edge, n** | 31 | 35 | 39 | 34 |
| None (score = 0) | 5 (16.1) | 7 (20.0) | 12 (30.8) | 5 (14.7) |
| Mild (score = 1) | 13 (41.9) | 14 (40.0) | 20 (51.3) | 17 (50.0) |
| Moderate (score = 2) | 11 (35.5) | 12 (34.3) | 5 (12.8) | 11 (32.4) |
| Severe (score = 3) | 2 (6.5) | 2 (5.7) | 2 (5.1) | 1 (2.9) |
| **PGI-I** |  |  |  |  |
| **ACROBAT Evolve, n** | − | − | 11 | 11 |
| Very much improved (score = −3) | − | − | 2 (18.2) | 0 (0.0) |
| Much improved (score = −2) | − | − | 3 (27.3) | 5 (45.5) |
| Minimally improved (score = −1) | − | − | 2 (18.2) | 0 (0.0) |
| No change (score = 0) | − | − | 4 (36.4) | 5 (45.5) |
| Minimally worse (score = 1) | − | − | 0 (0.0) | 1 (9.1) |
| Much worse (score = 2) | − | − | 0 (0.0) | 0 (0.0) |
| Very much worse (score = 3) | − | − | 0 (0.0) | 0 (0.0) |
| **ACROBAT Edge, n** |  |  | 36 | 38 |
| Very much improved (score = −3) | − | − | 2 (5.6) | 4 (10.5) |
| Much improved (score = −2) | − | − | 9 (25.0) | 5 (13.2) |
| Minimally improved (score = −1) | − | − | 10 (27.8) | 9 (23.7) |
| No change (score = 0) | − | − | 15 (41.7) | 16 (42.1) |
| Minimally worse (score = 1) | − | − | 0 (0.0) | 1 (2.6) |
| Much worse (score = 2) | − | − | 0 (0.0) | 3 (7.9) |
| Very much worse (score = 3) | − | − | 0 (0.0) | 0 (0.0) |

EOT = end of treatment at Week 13; PGI-I = Patient Global Impression of Improvement; PGI-S = Patient Global Impression of Severity.

'−' = Not administered. ^a^ Screening Visit 1b
